# Prevalence of Goiter and Thyroid Nodules before and after Implementation of the Universal Salt Iodization Program in Mainland China from 1985 to 2014: A Systematic Review and Meta-Analysis

**DOI:** 10.1371/journal.pone.0109549

**Published:** 2014-10-14

**Authors:** Wei Zhao, Cheng Han, Xiaoguang Shi, Chuhui Xiong, Jie Sun, Zhongyan Shan, Weiping Teng

**Affiliations:** Department of Endocrinology and Metabolism, Institute of Endocrinology, Liaoning Provincial Key Laboratory of Endocrine Diseases, the First Affiliated Hospital of China Medical University, Shenyang, Liaoning Province, People's Republic of China; National Institute for Viral Disease Control and Prevention, CDC, China, China

## Abstract

**Objectives:**

We comprehensively estimated the prevalence of goiter and thyroid nodules (TNs) before and after the implementation of the Universal Salt Iodization (USI) program in mainland China and provided information for creating effective health policies.

**Methods:**

PubMed, Google Scholar, CNKI, Chinese Wanfang and Chongqing VIP databases were searched for relevant studies from Jan 1985 to Feb 2014. Data from eligible citations were extracted by two independent reviewers. All analyses were performed with Stata 11.0 and SPSS 17.0.

**Results:**

Eligible articles (N = 31; 4 in English and 27 in Chinese) included 52 studies (15 about goiter rates made before 1996 and 14 afterwards, and 23 about TNs). Our meta-analysis suggests a pooled prevalence for goiter before and after 1996 and for TNs of 22.8% (95% CI: 15.3%, 30.3%), 12.6% (95% CI: 9.4%, 15.8%) and 22.7% (95% CI: 18.3%, 27.0%), respectively. Egger's test of three independent categories revealed no evidence of publication bias (*p* = 0.101, 0.148 and 0.113, respectively).

**Conclusions:**

The prevalence of goiter was reduced by almost half after 1996 in mainland China, so the USI program was considered beneficial. However, subgroup analysis suggests that both insufficient and excess iodine may be associated with goiter. The prevalence of goiter and TNs increased significantly after 2002, suggesting a risk of excessive iodine intake. Thus, salt iodization standardizations should be set according to local conditions.

## Introduction

Iodine is an essential trace element required for the normal thyroid hormone activity, specifically that of thyroxine and tri-iodothyronine. Both insufficient and excessive iodine intake can cause thyroid-hormone disorders [1]–[4] and the presence of goiter and thyroid nodules (TNs) represents thyroid diseases. The term *goiter* describes the enlargement of the thyroid gland and the goiter prevalence of this condition is considered an important and sensitive long-term indicator of iodine intake [1], [5]. The prevalence of goiter in school-age children is related to the severity of iodine deficiency. For example, a prevalence of 0.0–4.9% suggests no iodine deficiency; 5.0–19.9% indicates mild deficiency; 20.0–29.9% reveals moderate deficiency; and severe deficiency is observed at ≥30% [1]. Excessive iodine, however, can also lead to goiter [1].

TNs are discrete structurally distinct lesions within the thyroid gland, separate from the surrounding parenchyma [6]. Previous work indicates that the frequency of TNs vary considerably among the general population—4–7% are detected by palpation [7], 20–76% are found with ultrasound [8], [9], and 19–67% are documented from autopsy data [10]–[12]. Although most TNs are benign, cancer should be suspected with the presence of TNs [6], [13], because 5–15% may be malignant (carcinomas) [14]. Thus, goiter and TNs are common thyroid disorders with an intimate association with iodine intake.

China was once an iodine-deficient country; prior to the 1970s, 370 million people lived in iodine-deficient areas [15]. Iodine deficiencies manifested various ways, including goiter, cretinism, endemic mental retardation, and decreased fertility rates [1], all of which are generally classified as iodine deficiency disorders (IDDs) [1], [16]. To reduce IDDs, in 1979, a program of local iodine fortification was introduced into these iodine-deficient areas; and in 1996, China launched the Universal Salt Iodization (USI) program [17]. Although the USI program reduced the prevalence of goiter, the median urinary iodine concentration (UIC) in school-age children simultaneously rose sharply—reaching 330 µg/L in 1997 and 306 µg/L in 1999. For this reason, in 2002, national standards for iodized salt were revised to reduce the iodine concentration at the production level [5]. In 2012, global data identified China as a region with more than adequate iodine intake [18]. Meanwhile, since the implementation of the USI program, a growing number of Chinese clinical endocrinologists have reported an increasing incidence of thyroid diseases, especially in recent years [19]–[25].

China has diverse environments with varied populations and socio-economic conditions, complicating unified epidemiological investigations for prevalence of goiter and TNs, most of which were limited to specific geographic areas or populations. Thus, data may not accurately represent these two diseases especially in light of the implementation of the USI program which may skew data for the epidemiology of goiter and TNs. Thus, to formulate appropriate local public health policies and criteria, we must document the epidemiology and distribution of both diseases and use the few previous studies to provide comprehensive analyses of detailed explorations and secular trends, geographic properties, and iodine status.

Here, we describe a systematic review and meta-analysis of the prevalence of goiter and TNs before and after implementation of the USI program in mainland China from 1985 to 2014.

## Materials and Methods

### Search Strategy

Goiter and TNs are independent diseases and iodine intake in China changed markedly after 1996 after the implementation of USI. Therefore, we conducted three meta-analyses—one on the prevalence of goiter before 1996 and one after 1996 and one analysis of the prevalence of TNs. We searched all English-language reports of population-based studies on the prevalence of goiter and TNs using PubMed and Google Scholar, and searched all Chinese reports manually and on-line using the CNKI (Chinese National Knowledge Infrastructure), Chinese Wanfang and Chongqing VIP databases, from Jan 1985 to Feb 2014. Search key words were “goiter,” “thyroid nodule(s),” “thyroid disorder(s),” “epidemiology” and “prevalence(s)”. We also scanned relevant reference lists and reviews to find additional studies. Attempts were made to contact authors of the identified papers for necessary details not given in the original texts. The PRISMA guideline for systematic reviews and meta-analyses was followed closely [26], see Checklist S1.

### Inclusion and Exclusion Criteria

The following inclusion criteria were adopted for paper selection: (1) Data were acquired through population-based studies instead of hospital-based studies; (2) study participants were recruited from a random community-based sample, rather than from volunteers or those receiving routine-health examinations, and the subjects had to be living in mainland China; (3) the studies contained sufficient information to conduct pooled analysis of the prevalence; (4) if the same study data were reported in both English and Chinese, the English publication was included.

Studies were excluded if: (1) they were reviews or case reports; (2) the participants suffered from any related diseases or took medicines known to affect thyroid structure or function; (3) the study focused on participants with one underlying condition (such as pregnant women and smokers) or a certain occupation; (4) they were duplicate publications.

### Data Extraction

**Figure 1:**
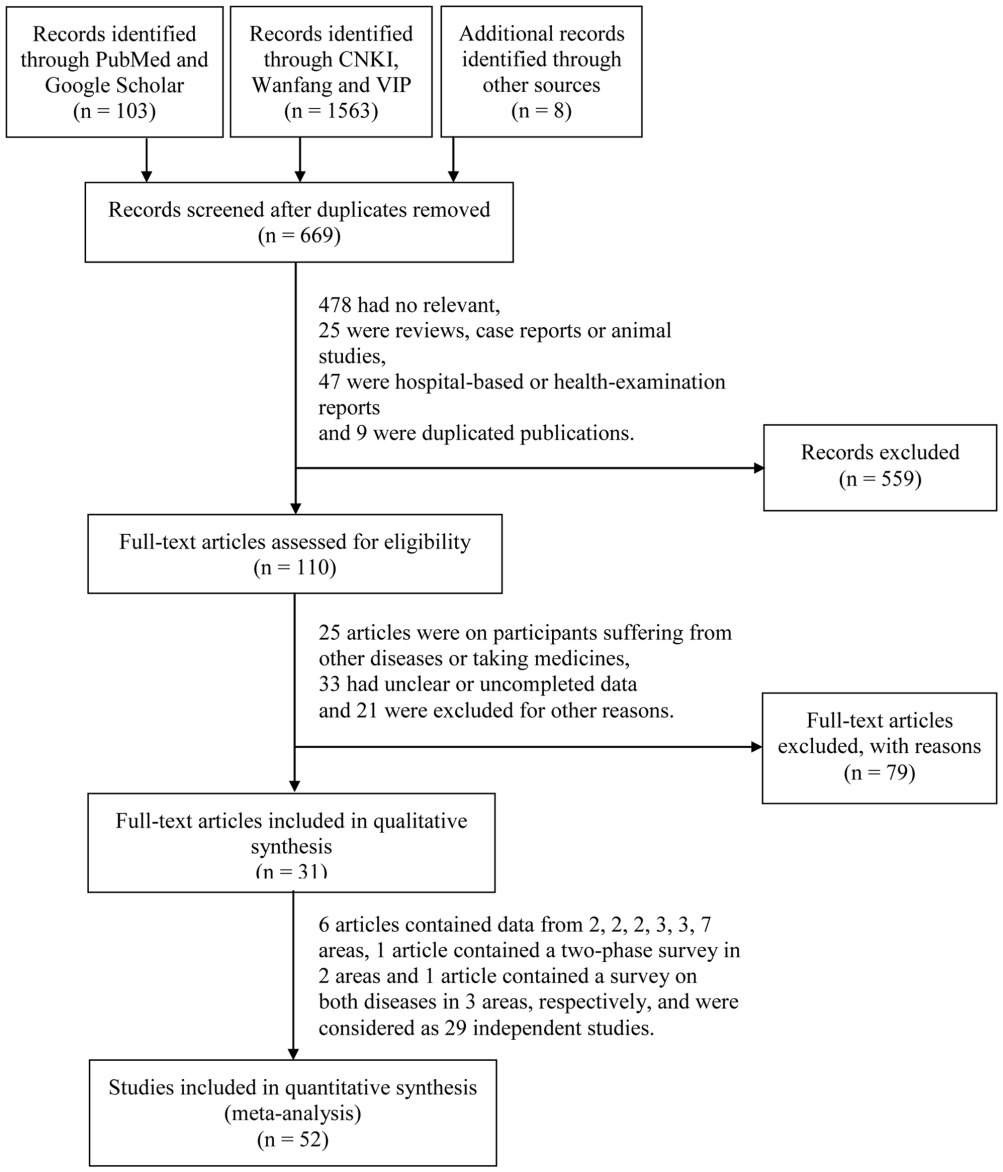
Flow diagram of the literature-search process.

The literature was searched independently by two reviewers. Any discrepancies between the extracted data of the two reviewers were reconciled through discussion. The literature-search process is shown in Figure 1. For all included studies, the first author's name, publication date, study year, age, location, sample size, events data and prevalence were recorded.

### Iodine Status

Median UIC is recognized as the most practical biochemical marker for iodine nutrition [27]. Using the criteria from WHO/UNICEF/ICCIDD [1], the iodine status was identified based on the median UIC, where the median UIC ≤99 µg/L is insufficient, 100–199 µg/L is adequate, 200–299 µg/L is more than adequate and a median UIC ≥300 µg/L is considered to be excessive.

### Statistical Analysis

The pooled prevalence and 95% confidence intervals (CIs) were used to estimate the prevalence of goiter and TNs in mainland China. All meta-analyses were calculated for heterogeneity using the Chi-square based Q test and the I^2^ test (25, 50 and 75% were considered low, moderate and high levels of heterogeneity, respectively) [28]. For a moderate or high level of heterogeneity we adopted a random-effects meta-analysis rather than using a fixed-effects model. Addressing heterogeneity and performing a secondary analysis required subgroup analysis. Publication bias was estimated through Egger's test. A *p*-value less than 0.05 indicated statistical significance. Meta-analyses were carried out in Stata Version 11.0 (Stata Corp LP, TX). Differences in prevalence among different groups were analyzed using the Chi-square test with SPSS Version 17.0 (SPSS Software, Chicago, IL).

## Results

### Characteristics of Papers

Figure 1 provides a schematic representation of the process for identifying, screening, and including studies in the review. The search strategy resulted in 31 articles (4 published in English and 27 in Chinese), reporting 52 studies—29 studies reporting goiter prevalence estimates (15 before 1996 and 14 after) and 23 on TNs. Table 1 provides a descriptive summary of these studies.

**Table 1:** Characteristics of studies on the prevalence of goiter and TNs.

First author	Publication year	Area	Rural/Urban	Inland/Coastal	Study year	Sample size	Case	Prevalence (%)
**Prevalence of goiter before 1996**								
Wang LF [29]	1992	Kunlun Plain, Xinjiang	Rural	Inland	1987	1236	629	50.89
Wang LF [29]	1992	Kunlun Plain, Xinjiang	Rural	Inland	1987	3686	1223	33.18
Wang LF [29]	1992	Kunlun Plain, Xinjiang	Rural	Inland	1987	2351	415	17.65
Dou DX [30]	1992	Zhaoguan, Anhui	Rural	Inland	1986	398	71	17.84
Chen HM [31]	2000	Puyang, Henan	Rural	Inland	1990	1589	336	21.15
Zhang JL [32]	1992	Linyi, Shandong	Mixed	Inland	1985	48083	2324	4.83
Zhang JL [32]	1992	Linyi, Shandong	Mixed	Inland	1989	110609	3445	3.11
Wu WY [33]	2002	Qingyuan, Zhejiang	Rural	Inland	1985	154451	60434	39.13
Zhang Z [34]	2002	Fuzhou, Fujian	Urban	Coastal	1989	10454	584	5.59
Zhang Z [34]	2002	Wuyishan, Fujian	Urban	Inland	1989	1032	253	24.52
Bao Y [35]	1994	Xuzhou, Jiangsu	Rural	Inland	1990	81548	16720	20.50
Bao Y [35]	1994	Xuzhou, Jiangsu	Urban	Inland	1990	3999	732	18.30
Su MY [36]	1989	Tekesi, Xinjiang	Rural	Inland	1987	202	92	45.54
Su MY [36]	1989	Tekesi, Xinjiang	Rural	Inland	1987	220	54	24.55
Zheng HB [37]	1988	Cangzhou, Hebei	Rural	Inland	1985	16324	2631	16.12
**Prevalence of goiter after 1996**								
Yu J [38]	2004	Huachuan, Heilongjiang	Rural	Inland	2002	112	21	18.75
Yu XH [39]	2008	Panshan, Liaoning	Rural	Inland	1999	815	189	23.19
Yu XH [39]	2008	Zhangwu, Liaoning	Rural	Inland	1999	1514	256	16.91
Yu XH [39]	2008	Huanghua, Hebei	Rural	Inland	1999	1056	80	7.58
Zhao SH [40]	2004	Yantai, Shandong	Rural	Coastal	2000	554	60	10.83
Zhao SH [40]	2004	Weihai, Shandong	Rural	Coastal	2000	543	54	9.94
Zhao SH [40]	2004	Weihai, Shandong	Rural	Coastal	2000	437	32	7.32
Zhao SH [40]	2004	Rizhao, Shandong	Rural	Coastal	2000	610	51	8.36
Zhao SH [40]	2004	Rizhao, Shandong	Rural	Coastal	2000	330	43	13.03
Zhao SH [40]	2004	Qingdao, Shandong	Rural	Coastal	2000	570	49	8.60
Zhao SH [40]	2004	Wulian, Shandong	Rural	Inland	2000	562	24	4.27
Zhang Z [34]	2002	Fuzhou, Fujian	Urban	Coastal	1999	3473	272	7. 83
Zhang Z [34]	2002	Wuyishan, Fujian	Urban	Inland	1999	2394	277	11.57
Zhu WY [41]	2009	Zhoushan, Zhejiang	Mixed	Coastal	2007	1389	421	30.31
**Prevalence of TNs**								
Yu XH [39]	2008	Panshan, Liaoning	Rural	Inland	1999	815	103	12.64
Yu XH [39]	2008	Zhangwu, Liaoning	Rural	Inland	1999	1514	154	10.17
Yu XH [39]	2008	Huanghua, Hebei	Rural	Inland	1999	1056	114	10.80
Chen ZX [42]	2013	Hangzhou, Zhejiang	Mixed	Inland	2010	9412	2822	29.98
Lou XM [43]	2011	Daishan, etc, Zhejiang	Mixed	Coastal	2009	456	66	14.47
Lou XM [43]	2011	Haining, etc, Zhejiang	Mixed	Coastal	2009	321	48	14.95
Lou XM [43]	2011	Xiangshan, etc, Zhejiang	Mixed	Coastal	2009	280	59	21.07
Feng SY [44]	2006	Gaochun and Chuzhou, Jiangsu	Rural	Inland	2005	2280	407	17.85
Li SJ [45]	2012	Fenghua, Zhejiang	Rural	Coastal	2010	781	160	20.49
Chi HY [46]	2013	Weihai, Shandong	Urban	Coastal	2008	4405	1390	31.56
Chen LD [47]	2011	Lishui, Zhejiang	Rural	Inland	2006	638	245	38.40
Wang XD [48]	2008	Wuxi, Jiangsu	Urban	Inland	2005	750	169	22.53
Liu F [49]	2012	Ruian, Zhejiang	Urban	Inland	2010	5060	1166	23.04
Yang NZ [50]	2012	Taizhou, Zhejiang	Mixed	Inland	2010	793	182	22.95
Liu ZY [51]	2012	Zhoushan, Zhejiang	Mixed	Coastal	2010	3206	926	28.88
Zhu WY [52]	2010	Zhoushan, Zhejiang	Mixed	Coastal	2006	3284	831	25.30
Zou SR [27]	2012	Shanghai	Mixed	Coastal	2009	7369	2011	27.29
Guo HW [53]	2014	Nanjing, Jiangsu	Urban	Inland	2011	9533	4439	46.56
Zhang JL [54]	2013	Yan'an, Shanxi	Mixed	Inland	2012	2970	822	27.68
Liu Y [55]	2012	Chengdu, Sichuan	Urban	Inland	2009	1500	255	17.00
Yang YX [56]	2011	Guiyang, Guizhou	Urban	Inland	2009	1512	153	10.12
Shen Y [57]	2013	Shanghai	Mixed	Coastal	2010	695	159	22.88
Zhang SG [58]	2010	Xuzhou, Jiangsu	Rural	Inland	2008	468	117	25.00

All studies were based on general population samples. Before 1996, 436,182 people met the inclusion criteria for goiter; the criteria for diagnosis were in line with those of WHO, which was based on palpations. Another 14,359 people met the criteria for goiter after 1996. Goiter diagnosis after 1996 was based on palpation or ultrasound. After a diagnose with ultrasound, 59,098 people met the inclusion criteria for TNs.

### Pooled Prevalence of Goiter and TNs

**Figure 2:**
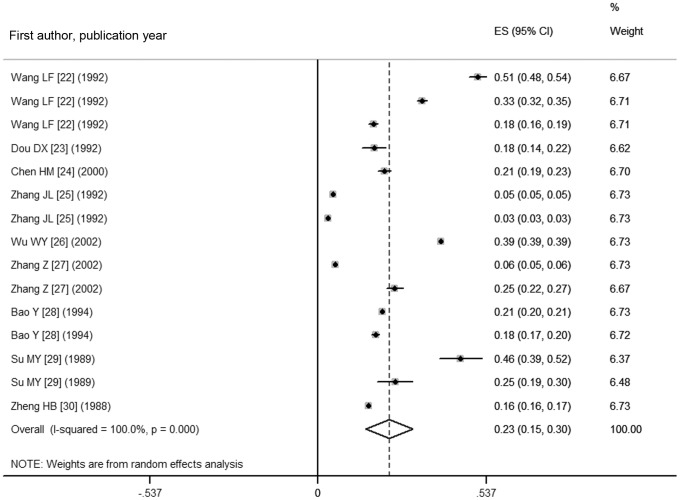
Forest plot displaying the pooled goiter prevalence in mainland China before 1996.

**Figure 3:**
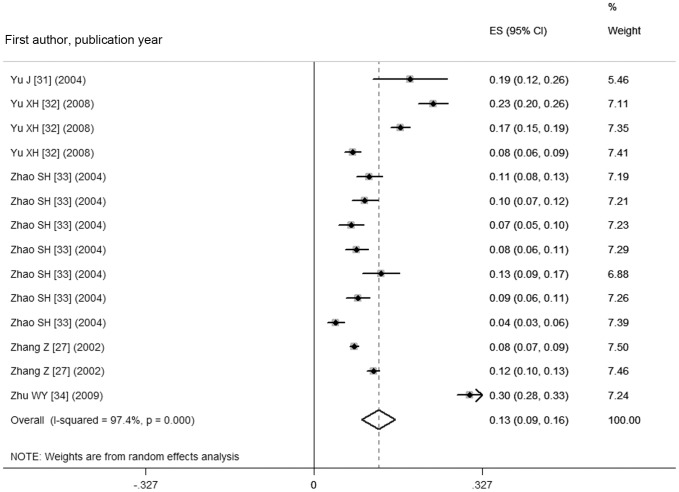
Forest plot displaying the pooled goiter prevalence in mainland China after 1996.

**Figure 4:**
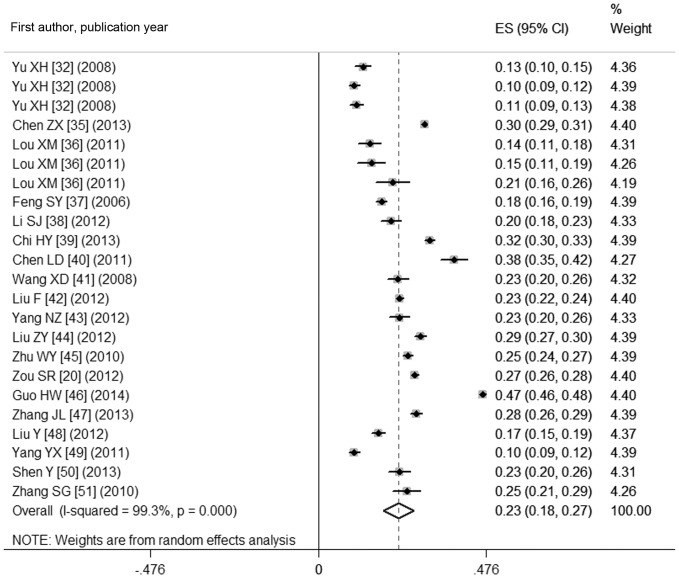
Forest plot of the pooled prevalence of TNs in mainland China.

**Figure 5:**
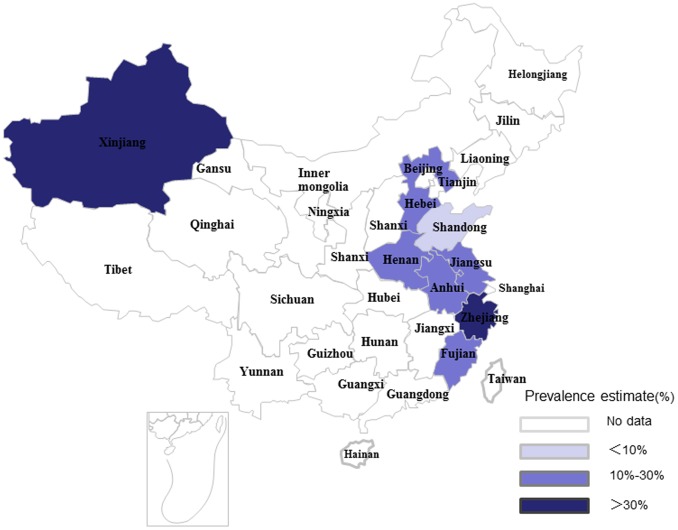
Regional distribution of pooled goiter prevalence in mainland China before 1996.

**Figure 6:**
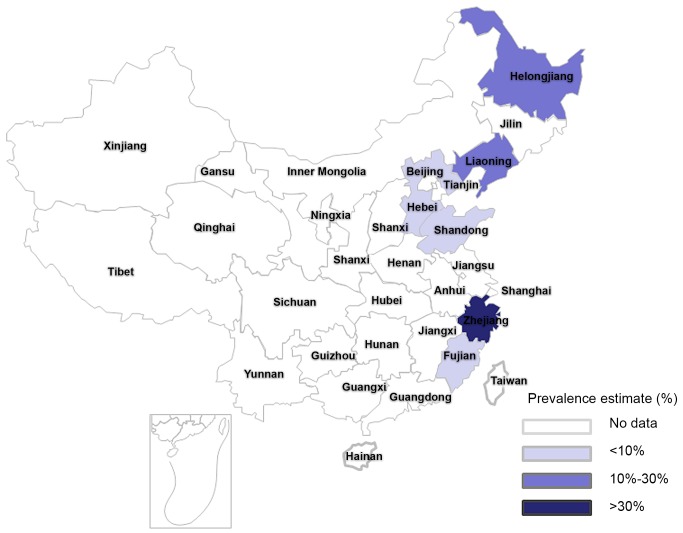
Regional distribution of pooled goiter prevalence in mainland China after 1996.

**Figure 7:**
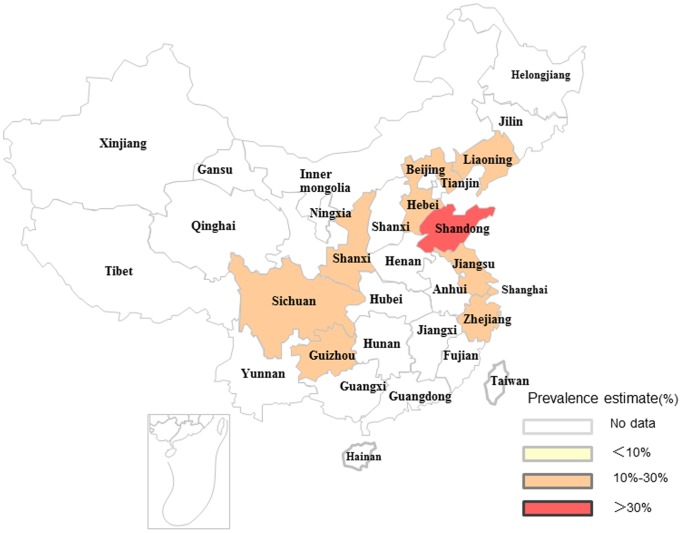
Regional distribution of pooled prevalence of TNs in mainland China.

As shown in Figs 2 and 3, the pooled prevalence of goiter before 1996 was 22.8% (95% CI: 15.3%, 30.3%), with the actual numbers ranging from 3.11 to 50.89%; after 1996, the pooled prevalence, 12.6% (95% CI: 9.4%, 15.8%) in the range of 4.27–30.31%, was significantly lower compared with the prevalence prior to 1996 (χ^2^ = 532.56, *p*<0.001). Figure 4 shows a pooled prevalence of 22.7% (95%CI: 18.3%, 27.0%) for TNs with individual studies ranging from 10.12–46.56%. Individual disease conditions of the provinces and municipalities are shown in the maps in Figs 5–7.

### Subgroup Analysis

Goiter prevalences before and after 1996 and TNs were analyzed in subgroups, divided by location (North, South, East, West and Central China) and the type of area (rural, urban and mixed), as shown in Tables 2–4.

**Table 2:** Prevalence of goiter before 1996 in mainland China by different stratification factors.

Subgroups	Prevalence % (95%CI)	No of studies	Heterogeneity		Case/Total
			I^2^%	*p* Value	
**Area**					
Rural	28.6 (20.7–36.5)	10	99.9	<0.001	82605/262005
Mixed	4.0 (2.3–5.7)	2	99.6	<0.001	5769/158692
Urban	16.1 (5.0–27.2)	3	99.6	<0.001	1569/15485
**Coastal/Inland**					
Coastal	5.6 (5.1–6.0)	1	-	-	584/10454
Inland	24.0 (16.0–32.1)	14	100.0	<0.001	89359/425728
**Altitude**					
<200	13.4 (8.3–18.5)	8	100.0	<0.001	26843/273004
200–500	31.9 (17.6–46.2)	2	99.2	<0.001	60687/155483
500–1000	-	0	-	-	-
>1000	34.3 (21.9–46.6)	5	99.2	<0.001	2413/7695
**Iodine Status**					
Insufficient	25.5 (12.0–39.1)	10	100.0	<0.001	67878/288418
Adequate	14.5 (−4.8–33.8)	2	97.8	<0.001	2378/48303
More than Adequate	-	0	-	-	-
Excess	19.2 (15.8–22.6)	3	98.9	<0.001	19687/99461
**Method**					
Palpation	22.8 (15.3–30.3)	15	100.0	<0.001	89943/436182
Ultrasound	-	0	-	-	-
**Sample Size**					
<5000	28.0 (21.3–34.8)	9	98.9	<0.001	3805/14713
>5000	14.9 (3.1–26.7)	6	100.0	<0.001	86138/421469
**Location**					
North China	16.1 (15.6–16.7)	1	-	-	2631/16324
South China	15.0 (−3.5–33.6)	2	99.5	<0.001	837/11486
East China	17.3 (4.8–29.8)	6	100.0	<0.001	83726/399088
West China	34.3 (21.9–46.6)	5	99.2	<0.001	2413/7695
Central China	21.1 (19.1–23.2)	1	-	-	336/1589
**Total**	**22.8 (15.3–30.3)**	**15**	**100.0**	**<0.001**	**89943/436182**

**Table 3:** Prevalence of goiter after 1996 in mainland China by different stratification factors.

Subgroups	Prevalence % (95%CI)	No of studies	Heterogeneity		Case/Total
			I^2^%	*p* Value	
**Area**					
Rural	11.5 (8.3–14.6)	11	95.0	<0.001	859/7103
Mixed	30.3 (27.9–32.7)	1	-	-	421/1389
Urban	9.7 (6.0–13.3)	2	95.5	<0.001	549/5867
**Coastal/Inland**					
Coastal	12.0 (7.1–16.9)	8	97.7	<0.001	982/7906
Inland	13.4 (8.5–18.4)	6	97.4	<0.001	847/6453
**Altitude**					
<200	13.4 (9.6–17.3)	12	97.5	<0.001	1528/11403
200–500	7.9 (0.8–15.1)	2	97.8	<0.001	301/2956
500–1000	-	0	-	-	-
>1000	-	0	-	-	-
**Iodine Status**					
Insufficient	23.2 (20.3–26.1)	1	-	-	189/815
Adequate	4.3 (2.6–5.9)	1	-	-	24/562
More than Adequate	9.8 (6.3–13.3)	5	94.7	<0.001	660/6604
Excess	14.5 (8.8–20.1)	7	97.7	<0.001	956/6378
**Method**					
Palpation	9.7 (6.0–13.3)	2	95.5	<0.001	549/5867
Ultrasound	13.2 (8.8–17.5)	12	97.6	<0.001	1280/8492
**Sample Size**					
<5000	12.6 (9.4–15.8)	14	97.4	<0.001	1829/14359
>5000	-	0	-	-	-
**Location**					
North China	16.5 (8.9–24.0)	4	97.3	<0.001	546/3497
South China	9.7 (6.0–13.3)	2	95.5	<0.001	549/5867
East China	11.6 (5.9–17.3)	8	97.9	<0.001	734/4995
West China	-	0	-	-	-
Central China	-	0	-	-	-
**Study Year**					
1996–2001	10.7 (8.3–13.1)	12	95.0	<0.001	1387/12858
2002–2014	25.1 (13.8–36.3)	2	88.7	0.003	442/1501
**Total**	**12.6 (9.4–15.8)**	**14**	**97.4**	**<0.001**	**1829/14359**

**Table 4:** Prevalence of TNs in mainland China by different stratification factors.

Subgroups	Prevalence % (95%CI)	No of studies	Heterogeneity		Case/Total
			I^2^%	*p* Value	
**Area**					
Rural	19.1 (13.7–24.6)	7	97.7	<0.001	1300/7552
Mixed	24.0 (21.6–26.4)	10	94.5	<0.001	7926/28786
Urban	25.1 (13.2–37.1)	6	99.8	<0.001	7572/22760
**Coastal/Inland**					
Coastal	23.3 (20.2–26.4)	9	95.5	<0.001	5650/20797
Inland	22.5 (15.7–29.2)	14	99.6	<0.001	11148/38301
**Altitude**					
<200	23.4 (18.7–28.0)	20	99.3	<0.001	15568/53116
200–500	17.0 (15.1–18.9)	1	-	-	255/1500
500–1000	-	0	-	-	-
>1000	18.9 (1.7–36.1)	2	99.6	-	975/4482
**Sample Size**					
<5000	20.7 (17.0–24.5)	19	98.4	<0.001	6360/27724
>5000	31.7 (21.8–41.7)	4	99.7	<0.001	10438/31374
**Location**					
North China	11.0 (9.7–12.3)	3	35.9	0.210	371/3385
South China	-	0	-	-	-
East China	25.5 (21.2–29.8)	17	99.1	<0.001	15197/49731
West China	18.3 (7.6–28.9)	3	99.2	<0.001	1230/5982
Central China	-	0	-	-	-
**Gender**					
Male	16.6 (12.1–21.2)	23	99.0	<0.001	5420/24469
Rural	11.1 (5.9–16.3)	7	97.3	<0.001	302/2708
Mixed	19.2 (16.6–21.7)	10	90.4	<0.001	2854/12865
Urban	19.2 (9.1–29.2)	6	99.3	<0.001	2264/8896
Female	26.9 (22.2–31.6)	23	99.0	<0.001	11378/34629
Rural	23.3 (17.5–29.0)	7	96.1	<0.001	998/4844
Mixed	28.5 (25.9–31.1)	10	90.1	<0.001	5072/15921
Urban	29.4 (16.6–42.2)	6	99.6	<0.001	5308/13864
**Study Year**					
1999–2001	11.0 (9.7–12.3)	3	35.9	0.210	371/3385
2002–2014	24.4 (20.2–28.7)	20	99.2	<0.001	16427/55713
**Total**	**22.7 (18.3–27.0)**	**23**	**99.3**	**<0.001**	**16798/59098**

As shown in Table 2, before 1996, rural area (χ^2^ = 48054.05, *p*<0.001), inland area (χ^2^ = 1479.00, *p*<0.001), high altitude (χ^2^ = 52150.24, *p*<0.001), small study sample size (χ^2^ = 255.52, *p*<0.001), location in West China (χ^2^ = 2023.41, *p*<0.001), and both low and high levels of iodine intake (χ^2^ = 8809.32, *p*<0.001) might indicate a high prevalence of goiter.

As shown in Table 3, after 1996, subgroups including rural area (χ^2^ = 448.82, *p*<0.001), diagnosis using ultrasound (χ^2^ = 101.98, *p*<0.001) and study year 2002–2014 (χ^2^ = 421.05, *p*<0.001) had a greater prevalence of goiter. Of note, iodine nutrition may also be associated with this greater prevalence. Among the four iodine intake categories, the prevalence of goiter was lowest in the subgroup with adequate iodine, 4.3% (95% CI: 2.6%, 5.9%), and prevalence data for the insufficient subgroup was 23.2% (95% CI: 20.3%, 26.1%) and for excess iodine subgroup was 14.5% (95% CI: 8.8%, 20.1%), both of which were higher (χ^2^ = 190.17, *p*<0.001).

For TNs, prevalence among subgroups of location and study year was very different (Table 4). Prevalence rates for North, East and West China were 11.0% (95% CI: 9.7%, 12.3%), 25.5% (95% CI: 21.2%, 29.8%) and 18.3% (95% CI: 7.6%, 28.9%) (χ^2^ = 800.56, *p*<0.001), respectively. In terms of the study year, 11.0% (95% CI: 9.7%, 12.3%) of people investigated between 1999 and 2001 were diagnosed with TNs and this increased to 24.4% (95% CI: 20.2%, 28.7%) after 2002 (χ^2^ = 538.27, *p*<0.001).

### Analysis of Heterogeneity and Publication Bias

We noted a significant overall heterogeneity within the studies (*p*<0.001, I^2^ = 97.4–100.0%), which decreased through subgroup analyses. Egger's test for the three independent research categories revealed no evidence of publication bias (*p* = 0.101, 0.148 and 0.113, respectively).

## Discussion

We examined 52 epidemiological research studies covering 14 provinces, municipalities and autonomous regions in China. We systematically analyzed the prevalence of goiter and TNs prior to and after 1996, when China implemented the USI program. To our knowledge this is one of the first studies of this kind.

Research indicates that the prevalence of goiter in children 8–10 years-of-age is an indicator of local iodine consumption [1]. Our analysis, however, focused on the general population, to provide epidemiological information concerning the disease itself. Iodine nutrition is certainly an important factor for goiter, and the association between iodine intake and goiter prevalence has been investigated extensively.

Indian researchers reported that the prevalence of goiter was 65.9% in an iodine-deficient area [59], which decreased to 27.7% two decades after the USI program [60]. In Denmark, the goiter prevalence was 13.4% in an area with mild iodine deficiency and 19.7% in an area with moderate iodine deficiency, indicating that even a modest increase in iodine intake might significantly reduce IDDs [61]. In China, the USI program was launched in 1996 to prevent IDDs, establishing an important time boundary for our study. We observed a trend similar to the study in India. The overall goiter prevalence was 22.8% prior to 1996 and this was halved (declined to 12.6%). Thus, the USI program might be beneficial in China.

Also, iodine excess may increase thyroid volume [1]. In our subgroup analyses, both before and after 1996, the prevalence of goiter significantly changed with iodine status, with data suggesting hormesis—emphasizing that overdoses of iodine, as well as deficiencies may be associated with high prevalence of goiter. Meanwhile, previous studies suggest a goitrogenic effect of excess iodine in school-age children and adults [62]–[67]. In areas with high iodine content in the drinking water, school-age children had elevated median UIC and endemic goiter, suggesting that, in addition to adjusting the iodine content of salt, improving water quality is also necessary [62], [63]. In a prospective study, 10 normal men accepted daily oral intake of excess iodine for 1 month. At the end of this treatment, their mean thyroid volume increased, which eventually returned to baseline within 4 weeks after iodine withdrawal [64]. LeMar reported that excess iodine from tetraglycine hydroperiodide tablets caused a reversible thyroid stimulating hormone (TSH)-dependent thyroid enlargement [65]. For Peace Corps volunteers, after the removal of excess iodine from water, the mean serum iodine declined sharply, and the goiter rate fell from 44 to 30% [66]. In animal models, excessive iodine intake has been shown to lead to thyroid enlargement [67]. Thus, iodine excess should be scrutinized. In agreement with this concern, we observed that after 1996, the pooled goiter prevalence increased from 10.7% before 2002 to 25.1% afterwards, indicating an impending problem.

We also observed that altitude correlated with goiter: the plateau environment was associated with higher goiter frequency than residence in the plains and hills, a finding similar to trends in previous surveys that may be explained by deficiencies in natural iodine at high altitudes and limited educational and economic support for the people who reside there [68]–[70].

There is a longstanding controversy regarding the relationship between the prevalence of TNs and iodine nutrition because of the varied epidemiological situation across the globe. Among a Swedish population with adequate iodine, the rate of TNs was only 2.6% [71]. German data from a previously iodine deficient area revealed a 20.2% prevalence of TNs, with a smaller rate for men [72]. Mexico, which formerly was mildly iodine deficient, now consumes more than normal iodine intake and here the locally-identified TNs frequency was 19.6% [73]. Overall, the prevalence of TNs in mainland China was similar to that of other countries and regions of historic iodine deficiency and present states of iodine sufficiency. In addition, our data indicate that the prevalence of TNs after 2002 was higher than the rate prior to 2002—11.0% of those investigated from 1999–2001 were diagnosed with TNs, and this increased to 24.4% between 2002 and 2014.

Unlike goiter, which has a strong etiological relationship with iodine nutrition, the prevalence of TNs also depends on sex, age and head-and-neck radiation exposure history [74], [75]. According to the present information, TNs are more common in cities than in the country, which may be explained by lifestyle choices [76], [77]. Furthermore, females were at greater risk for TNs and this difference was not changed when females were further divided into rural, mixed and urban groups.

In conclusion, we report that the USI program in China successfully reduced the prevalence of goiter after 1996. However, the program might cause excesses iodine intake that may be associated with a high prevalence of goiter. The prevalence of TNs also increased over time. Also, 5% of TNs may be malignant [14], so an increased prevalence of TNs might predict more thyroid carcinoma patients. In view of this information, in 2012, China adjusted the iodized salt concentration to 20–30 mg/kg (previously 35 mg/kg) and provincial governments and health administrative departments were allowed to formulate local standards within ±30% of recommended concentrations based on data for their area [78], [79].

Our study has several limitations. First, variations in the quality of the selected papers exist. Uncertain data will confound investigations into potential influences on heterogeneity. Secondly, primary TNs articles did not use a completely unified diagnostic criterion, but they did diagnose with ultrasound. Third, data from qualified articles failed to cover most of mainland China provinces. Still, we included all available information about goiter and TNs from the past three decades and this report is the first to document the epidemiological status of both diseases before and after the USI program in mainland China. In addition, our work underscores the need for additional population-based studies in areas excluded from this analysis.

Our data show that the implementation of USI program is beneficial but may pose iodine risks; therefore, salt iodization standards should be set according to local conditions. Also, some epidemiology studies remain to be undertaken, and these are essential for comprehensive original data on the epidemiology and distribution of thyroid diseases. Our future work will include a national baseline study on thyroid diseases which will be implemented from 2014 to 2016 and these data will provide broad and accurate information for other researchers.

## Supporting Information

Checklist S1 **PRISMA 2009 Checklist.**
(DOC)
